# The COVID-19 Clinical Spectrum and the Effect of Associated Comorbidities on Illness Severity in the North Indian Population: A Cross-Sectional Study

**DOI:** 10.7759/cureus.28192

**Published:** 2022-08-20

**Authors:** Mahak Lamba, Praveen Raman Mishra, Rahul Verma, Akash Bharti, VPS Punia, Shaavi Mittal

**Affiliations:** 1 Internal Medicine, King George's Medical University, Lucknow, IND; 2 Internal Medicine, All India Institute of Medical Sciences, Gorakhpur, IND; 3 Respiratory Medicine, School of Medical Sciences & Research, Sharda University, Greater Noida, IND; 4 Internal Medicine, School of Medical Sciences & Research, Sharda University, Greater Noida, IND

**Keywords:** coronavirus disease 2019, covid-19, comorbidities, coronary artery disease, chronic kidney disease, ckd, cad, hypertension, diabetes, severity

## Abstract

Background: The activity level of the 2019 novel coronavirus (2019-nCoV) or coronavirus disease 2019 (COVID-19), as it is now called, is considered low. Despite early preventive lockdown measures and a massive vaccination drive, almost the entire adult population in India will have been vaccinated at least once by the beginning of 2022 (2,072,946,593 till 11 August 2022). There is still concern about a pan-India outbreak and threat due to newly emerging pathogenic strains. The goal of this study is to find out how common various presenting complaints are in COVID-19 patients as well as how comorbidities affect the severity of the illness.

Methods: This cross-sectional observational study was conducted from December 2020 to January 2021 at a tertiary care hospital's department of internal medicine in North India. The study included 237 patients who were COVID-19-positive and were admitted to our hospital after providing informed consent. They were classified into three groups: mild, moderate, and severe.

Results: Fever was the most common presenting symptom, affecting 84.4% of the population, while diarrhoea was the least common, affecting only 3.4% of the population. Fever, cough, sore throat, headache, and breathlessness were significantly correlated with the severity of the illness. Gastrointestinal symptoms like diarrhoea did not have any significant correlation with the severity of the illness. The severity of illness was statistically related to comorbidities such as hypertension, diabetes, coronary artery disease, chronic kidney disease, and chronic obstructive pulmonary disease.

Conclusion: Males were more likely to develop more serious illnesses. However, the correlation was not statistically significant. The number of comorbid conditions and the severity of the illness were found to have a fair and significant relationship. None of the diarrhoea symptoms were related to the severity of the illness.

## Introduction

The 2019 novel coronavirus (2019-nCoV) or coronavirus disease 2019 (COVID-19), as it is now called, is rapidly spreading worldwide from its place of origin in Wuhan city of Hubei province of China [1]. The 2019-nCoV has a close similarity to bat coronaviruses, and it has been postulated that bats are the primary source. While the origin of the 2019-nCoV is still being investigated, current evidence suggests that spread to humans occurred via transmission from wild animals illegally sold in the Huanan Seafood Wholesale Market [2]. The pandemic has been renamed SARS-CoV-2 (severe acute respiratory syndrome coronavirus 2) due to its resemblance to severe acute respiratory syndrome [3,4]. The WHO declared COVID-19 a global pandemic on 11th March 2020 [5].

The severe acute respiratory syndrome outbreak was encountered in 2003, and, more recently, there was an outbreak of a related but different coronavirus, the Middle East respiratory syndrome coronavirus [6]. The primary coronavirus, discovered in bats in 1937, rarely infects humans and is found in animals such as bats, camels, and cats rather than humans or other mammals. They later mutated, causing the disease to infect rats, cows, pigs, mice, cats, dogs, horses, and turkeys. Cough and cold were common symptoms of the first human coronavirus discovered in the 1960s [7].

The enormity of symptoms and clinical manifestations observed in COVID-19 patients displayed a vast array of inter-individual differences. According to a meta-analysis by Sanyaolu et al., conducted in 2020, the most common symptom during COVID-19 was fever followed by dry cough [8]. It has been observed that most of the severely affected patients also had pre-existing comorbidities such as diabetes, cardiovascular conditions, and hypertension [8]. It was displayed that a higher probability of getting admitted to intensive care units also had a high mortality rate [9,10].

It is therefore required to evaluate the symptoms, mortality rates, patient profiles, and severity of the illness in the pre-vaccination era to assess early detection and appropriate treatment of critical cases. This is an invaluable asset to the healthcare system in India, which displays immense socio-economic and cultural diversity. Accordingly, this study aims to understand the prevalence of symptoms, comorbidities, and mortality rates in the pre-vaccination era and how they can be utilized to prevent future COVID-19 outbreaks.

## Materials and methods

This cross-sectional observational study lasted 62 days, from December 2020 to January 2021. The study focused on COVID-19 cases admitted to Sharda Hospital in Greater Noida, Uttar Pradesh, India. All patients diagnosed with COVID-19 admitted to Sharda Hospital and willing to participate in this study were enrolled in the study. Patients under the age of 13 and those who refused to participate were excluded from the study.

The prevalence of fever was found to be 88.7% during hospitalization among diagnosed cases of COVID-19, according to a study by Guan et al. [11]. The sample size was calculated using the formula: n = Z^2^pq/d^2^; where Z is the ordinate of standard normal distribution at α% level of significance (1.96 at α = 5% level of significance), p is the observed prevalence, q = 100 - p, and d is the margin of error (5%). According to the calculation, 154 subjects were required for the study. During the study, we were able to recruit 237 patients for the study.

All cases of COVID-19 infections underwent the nasopharyngeal swab test, which was evaluated using reverse transcription polymerase chain reaction for the presence of the COVID-19 virus. All the patients included in the study underwent a thorough history taking, which included demographic parameters such as age, gender, and area of residence, i.e., urban or rural, and a rigorous evaluation for detection of various symptoms of COVID-19 infections such as fever, chills/rigour, cough, fatigue, anorexia, myalgia, sore throat, and rhinorrhea. Other comorbid illnesses such as cardiovascular disease, diabetes mellitus, hypertension, chronic lung disease, chronic kidney disease, cancer, and obesity were also evaluated in all cases. Patients were classified based on the severity of symptoms [12], as shown in Table 1.

**Table 1:** Severity of symptoms SpO2: oxygen saturation; PaO2: partial pressure of oxygen; FiO2: fraction of inspired oxygen; MODS: multiple organ dysfunction syndrome.

Severity of symptoms
Mild: low-grade fever, nausea, vomiting, and diarrhoea, no alteration in mental status, and immunocompetent
Moderate: respiratory rate greater than 30 breaths per minute, SpO2 greater than 93%, PaO2/FiO2 greater than 300, and pulmonary infiltrates greater than 50% within 24 to 48 hours
Severe: septic shock and MODS are all examples of critical respiratory failure (requiring mechanical ventilation)

Laboratory investigations included chest X-ray, complete blood count, renal function test, liver function test, electrocardiography, blood sugar, and body mass index calculation.

Statistical analysis

Data were gathered and tabulated using Microsoft Excel 2007 (Microsoft Corporation, Redmond, WA) and analysed using the statistical software package SPSS version 26.0 (IBM Corp., Armonk, NY). The mean and standard deviation (SD) of the quantitative parametric data were presented. Percentages and proportions were used to represent qualitative data. As a means of determining significance, the chi-square test was used to compare categorical variables. Other descriptive statistics like ANOVA and independent t-test were employed to compare continuous data. A p-value of <0.05 was considered to be statistically significant.

## Results

The mean ± SD of age among the study participants was 37.89 ± 16.10 years. Maximum subjects were between the age group of 21-30 years (32.1%), followed by 31-40 years (19.8%) and 41-50 years (14.8%). Approximately 14.3% of the subjects were asymptomatic. Of the patients, 84.4% had a fever, 59.9% had a cough, 40.5% had a sore throat, 21.5% had a headache, 26.6% had breathlessness, and 3.4% had diarrhoea. Comorbidities, namely, hypertension, diabetes, coronary artery disease, chronic kidney disease, and chronic obstructive pulmonary disease (COPD) accounted for 8.4%, 6.8%, 2.1%, 2.5%, and 3.4% of the study population, respectively (Table 2).

**Table 2:** Distribution of demographic variables among the study participants

Variable	Frequency	Percentage
Age group (in years)		
10-20	25	10.5
21-30	76	32.1
31-40	47	19.8
41-50	35	14.8
51-60	32	13.5
61-70	10	4.2
71-80	9	3.8
>80	3	1.3
Gender		
Male	155	65.4
Female	82	34.6
Asymptomatic		
Yes	34	14.3
No	203	85.7
Fever		
Yes	200	84.4
No	37	15.6
Cough		
Yes	142	59.9
No	95	40.1
Sore throat		
Yes	96	40.5
No	141	59.5
Headache		
Yes	51	21.5
No	186	78.5
Breathlessness		
Yes	63	26.6
No	174	73.4
Diarrhoea		
Yes	8	3.4
No	229	96.6
Acute respiratory distress syndrome		
Yes	24	10.1
No	213	89.9

The distribution of comorbidities was found to be the same in asymptomatic and symptomatic individuals. When different comorbidities were compared between the asymptomatic and symptomatic groups using the chi-square test, an insignificant difference was found at p > 0.05 (Table 3).

**Table 3:** Distribution of comorbidities with symptoms of the study participants

Comorbidities	Asymptomatic (n = 34), N (%)	Symptomatic (n = 203), N (%)	P-value
Hypertension			
Present	1 (2.9%)	19 (9.36%)	0.21
Absent	33 (97.1%)	184 (90.64%)	
Diabetes mellitus			
Present	0	16 (7.9%)	0.09
Absent	34 (100%)	187 (92.1%)	
Coronary artery disease			
Present	0	5 (2.5%)	0.355
Absent	34 (100%)	198 (97.5%)	
Chronic kidney disease			
Present	1 (2.9%)	5 (2.5%)	0.87
Absent	33 (97.1%)	198 (97.5%)	
Chronic obstructive pulmonary disease			
Present	0	8 (3.9%)	0.24
Absent	34 (100%)	195 (96.1%)	

**Figure 1:**
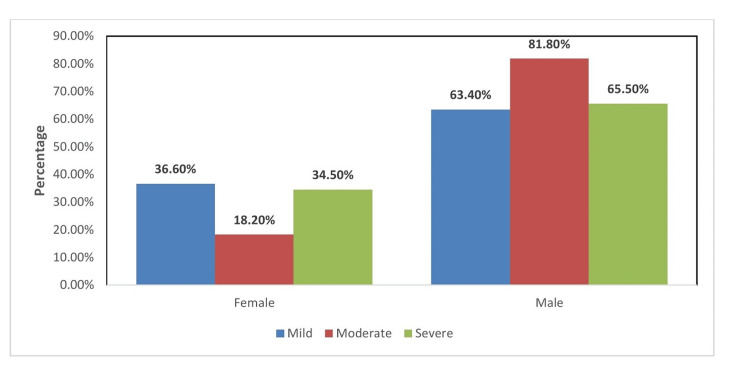
Distribution of gender with the severity of illness among the study participants

Mild, moderate, and severe illness was found among 68, 4, and 10 females and 118, 18, and 19 males, respectively. Hence, the severity of illness was found to be more among males as compared to females, as shown in Figure 1.

**Figure 2:**
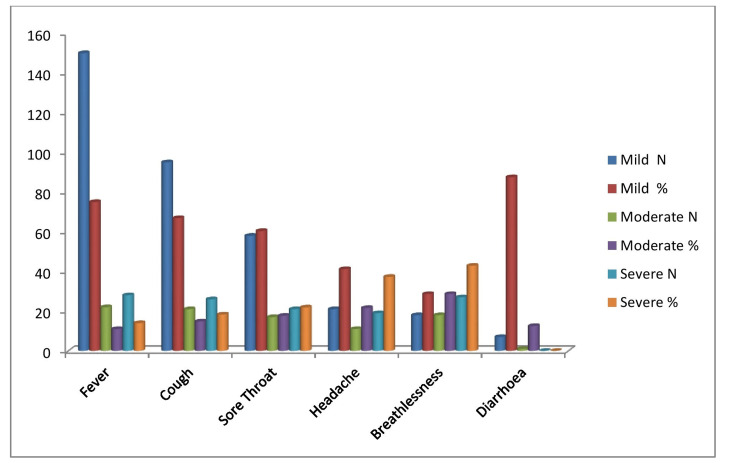
Distribution of symptoms with the severity of illness among the study participants

Fever, cough, sore throat, headache, and breathlessness were significantly correlated with the severity of the illness. Gastrointestinal symptoms like diarrhoea did not have any significant correlation with the severity of the illness (Figure 2).

The severity of illness was statistically related to comorbidities such as hypertension, diabetes, coronary artery disease, chronic kidney disease, and COPD. Patients with severe illness had a higher prevalence of hypertension than those with mild or moderate illness (p = 0.001). Patients with severe illness had a higher prevalence of diabetes than those with mild or moderate illness (p = 0.001). Patients with severe illnesses had a higher prevalence of coronary artery disease than patients with mild or moderate illness (p = 0.002). Patients with severe illness had a higher prevalence of chronic kidney disease than patients with mild illness (p = 0.001), as shown in Table 4.

**Table 4:** Distribution of comorbidities with the severity of illness among the study participants

Comorbidities	Mild, N (%)	Moderate, N (%)	Severe, N (%)	P-value
Hypertension				
Present	4 (2.2%)	5 (22.7%)	11 (37.9%)	<0.001
Absent	182 (97.8%)	17 (77.3%)	18 (62.1%)	
Diabetes mellitus				
Present	6 (3.2%)	2 (9.1%)	8 (27.6%)	<0.001
Absent	180 (96.8%)	20 (90.9%)	21 (72.4%)	
Coronary artery disease				
Present	1 (0.5%)	1 (4.5%)	3 (10.3%)	0.002
Absent	185 (99.5%)	21 (95.5%)	26 (89.7%)	
Chronic kidney disease				
Present	1 (0.5%)	0	5 (17.2%)	<0.001
Absent	185 (99.5%)	22 (100%)	24 (82.8%)	
Chronic obstructive pulmonary disease				
Present	2 (1.1%)	1 (4.5%)	5 (17.2%)	<0.001
Absent	184 (98.9%)	21 (95.5%)	24 (82.8%)	

All laboratory parameters evaluated were found to be elevated in patients suffering from a severe illness as compared to mild and moderate illness (Table 5).

**Table 5:** Distribution of laboratory parameters with the severity of illness among the study participants CRP: C-reactive protein; IL-6: interleukin-6; SPO2: oxygen saturation; RR: respiratory rate; SGPT: serum glutamic pyruvic transaminase; SGOT: serum glutamic oxaloacetic transaminase; TLC: total leucocyte count; CTSS: CT severity score.

Laboratory parameters	Mild (mean ± SD)	Moderate (mean ± SD)	Severe (mean ± SD)	P-value
D-dimer	0.89 ± 0.88	3.69 ± 2.10	7.44 ± 2.72	<0.001
CRP	62.95 ± 18.09	84.73 ± 29.41	109.07 ± 40.42	<0.001
Ferritin	229.09 ± 62.24	564.23 ± 150.42	870.41 ± 130.59	<0.001
IL-6	4.47 ± 3.30	17.18 ± 8.43	41.52 ± 7.68	<0.001
SPO_2_	0.98 ± 0.13	0.94 ± 0.02	0.91 ± 0.03	0.52
RR	18.97 ± 2.73	22.32 ± 2.95	25.45 ± 3.54	<0.001
SGPT	49.80 ± 5.79	72.09 ± 24.11	132.07 ± 52.34	<0.001
SGOT	55.65 ± 5.69	77.09 ± 25.45	139.45 ± 53.58	<0.001
TLC	12170.97 ± 2417.86	14120.91 ± 4440.43	15757.93 ± 4243.20	<0.001
CTSS	3.02 ± 1.91	9.82 ± 1.79	19.00 ± 2.86	<0.001

C-reactive protein (CRP), ferritin, interleukin-6 (IL-6), respiratory rate (RR), serum glutamic pyruvic transaminase (SGPT), serum glutamic oxaloacetic transaminase (SGOT), total leucocyte count (TLC), and CT severity score (CTSS) were all found to be significantly higher in symptomatic patients as compared to asymptomatic patients (Table 6). To calculate CTSS, according to the anatomic structure, 18 segments of both lungs were divided into 20 regions, in which the posterior apical segment of the left upper lobe was subdivided into apical and posterior segmental regions, whereas the anteromedial basal segment of the left lower lobe was subdivided into anterior and basal segmental regions. The lung opacities in all of the 20 lung regions were subjectively evaluated on chest CT images using a system attributing scores of 0, 1, and 2 if parenchymal opacification involved 0%, less than 50%, or equal to or more than 50% of each region, respectively. The CTSS was defined as the sum of the individual scores in the 20 lung segment regions, which may range from 0 to 40 points [13].

**Table 6:** Distribution of laboratory parameters with asymptomatic individuals among the study participants CRP: C-reactive protein; IL-6: interleukin-6; SPO2: oxygen saturation; RR: respiratory rate; SGPT: serum glutamic pyruvic transaminase; SGOT: serum glutamic oxaloacetic transaminase; TLC: total leucocyte count; CTSS: CT severity score.

Laboratory parameters	Asymptomatic (n = 34)	Symptomatic (n = 203)	P-value
D-dimer	0.63 ± 0.75	2.19 ± 2.74	<0.001
CRP	56.03 ± 9.79	73.05 ± 29.10	<0.001
Ferritin	226.41 ± 119.32	357.48 ± 246.27	<0.001
IL-6	3.03 ± 5.51	11.38 ± 13.67	<0.001
SPO_2_	0.98 ± 0.008	0.96 ± 0.19	0.52
RR	17.53 ± 1.56	20.50 ± 3.69	<0.001
SGPT	47.21 ± 5.08	64.40 ± 35.73	<0.001
SGOT	53.12 ± 5.12	70.36 ± 36.48	<0.001
TLC	9805.88 ± 2057.35	13290.84 ± 3044.26	<0.001
CTSS	3.02 ± 1.91	6.39 ± 1.57	<0.001

## Discussion

The clinical and epidemiological characteristics of 237 COVID-19 patients in the tropical region of North India reveal a variety of patterns that can aid in the management of COVID-19 patients and provide a platform for developing strategies for improving public health strategies, which could help us to flatten the epidemic curve and decelerate COVID-19 disease explosion. The majority of the patients in the sample (158, 66.7%) were found to be the most severely affected, with the majority of them being between the ages of 20 and 49 years. In this study, the severity of illness was reported more in males as compared to females. Similar male dominance was revealed by Singh et al. [14].

During the study period, no home quarantine was permitted in Uttar Pradesh, so the majority of patients were admitted to a quarantine centre or a COVID-19-dedicated hospital. In this study, the most common symptom was fever, followed by cough and sore throat. Similarly, Guan et al. reported fever (88.7%) as the most common symptom [11]. Our study had a higher prevalence of fever and cough compared to Singh et al. but a lower percentage of fever than those reported by Wang et al. [14,15]. The presence of similar signs and symptoms in both the temperate Wuhan region of China and the tropical region of India lends credence to the idea that these signs and symptoms could be used as a first public screening measure and tool to test for SARS-CoV-2 infection suspects.

Of our study participants, 8.4% had hypertension, 6.8% had diabetes, 2.1% had coronary artery disease, 2.5% had chronic kidney disease, and 3.4% had COPD. Approximately similar findings were revealed by Singh et al. [14]. Diabetes was linked to a two-fold increase in COVID-19 mortality and severity compared to non-diabetics in Kumar et al.'s study [16]. According to Singh et al., the most common comorbidities associated with mortality were hypertension and chronic kidney disease [14].

A population-based surveillance report via COVID-19-Associated Hospitalization Surveillance Network reported clinical data on 1478 COVID-19-positive patient hospitalizations. Among the 1478 patients studied, 12% of adults showed clinical data of underlying medical conditions with the most prevalent being hypertension (49.7%) and obesity a close second (48.3%). Other medical conditions included chronic lung disease (34.6%), diabetes mellitus (28.3%), and cardiovascular diseases (27.8%) [17]. This was similar to our study. In a study by Filardo et al., advanced age, male sex, and obesity were the main factors associated with mortality. Cardiovascular and renal comorbidities were not associated with mortality in an adjusted analysis, perhaps owing to the limited sample size and the inability to detect these associations [18].

Due to COVID-19 being a relatively new disease, the data available were limited. However, from the cases that emerged, it was observed that comorbidities increase the chances of infection. Based on current information and clinical expertise, a generalization can be made that those with comorbidities have more symptomatic COVID. The elderly, a vulnerable population, with chronic health conditions such as diabetes and cardiovascular or lung disease are not only at a higher risk of developing severe illness but are also at an increased risk of death if they become ill. People with underlying uncontrolled medical conditions such as diabetes, hypertension, lung, liver, and kidney disease, and patients taking steroids chronically are at increased risk of COVID-19 infection [11,14].

In this study, IL-6 was found to be significantly more in symptomatic as compared to asymptomatic patients. Chen et al. reported a similar finding with significant elevation in the level of inflammatory cytokine IL‑6 in critically ill COVID‑19 patients [2]. Similarly, Liu et al. showed that a higher serum level of IL-6 was an independent and reliable risk factor for COVID-19 patients and led to higher disease severity and mortality [19]. IL‑6 is an important pro‑inflammatory factor in the disease process of SARS‑CoV‑2. It contributes to COVID‑19‑associated cytokine storms, largely enhancing vascular permeability and impairing organ function. The SARS‑CoV‑2 virus replicates rapidly, triggering a storm characterized by increased levels of cytokines such as IL‑6. Such an inflammatory response causes inflammation of the respiratory system and other bodily systems, with subsequent occurrence of acute respiratory distress syndrome or respiratory failure. Thus, estimation of IL‑6 levels could be an important tool to assess disease severity in COVID‑19 patients.

CTSS was found to be significantly more among symptomatic as compared to asymptomatic patients in this study. In a study by Raoufi et al., patients with lower CTSS had lower mortality [20]. According to Zayed et al., chest CT plays a segregate role in COVID-19 disease, adds an advantage in clinical data in triage, and highlights the decision of hospital admission [21].

Limitations

The major limitations of our study were that the prevalence of comorbidities was lower at an earlier age, and the sample population with comorbidities was small. Despite the study's inclusion of a sufficient number of COVID-19 patients, no significant associations between multiple comorbidities and mortality were found. The population was unvaccinated as India began vaccination on 16 January 2021.

Strength

The unique aspect of our study was the comparison of interleukin and CTSS scores among asymptomatic and symptomatic patients in a resource-limited setting.

## Conclusions

Males were more likely to develop more serious illnesses. The number of comorbid conditions and the severity of the illness were found to have a significant relationship. Fever, cough, sore throat, headache, and shortness of breath were found to be associated with a higher degree of illness. None of the diarrhoea symptoms were related to the severity of the illness. Hypertension, diabetes, coronary artery disease, chronic kidney disease, and COPD were all found to have statistically significant associations with illness severity.
